# Investigation of *SIRT1* gene variants in HIV-associated lipodystrophy and metabolic syndrome

**DOI:** 10.1590/1678-4685-GMB-2019-0142

**Published:** 2020-02-14

**Authors:** Carmela Farias da Silva Tagliari, Cáren Nunes de Oliveira, Greice Meyer Vogel, Patrícia Baptista da Silva, Rafael Linden, Rosmeri Kuhmmer Lazzaretti, Regina Kuhmmer Notti, Eduardo Sprinz, Vanessa Suñé Mattevi

**Affiliations:** ^1^ Universidade Federal de Ciências da Saúde de Porto Alegre (UFCSPA), Laboratório de Biologia Molecular, Porto Alegre, RS, Brazil.; ^2^ Universidade Feevale, Instituto de Ciências da Saúde, Laboratório de Toxicologia Analítica, Novo Hamburgo, RS, Brazil.; ^3^ Universidade Federal do Rio Grande do Sul, Hospital de Clínicas de Porto Alegre, Departamento de Doenças Infecciosas, Porto Alegre, RS, Brazil.

**Keywords:** HIV, lipodystrophy, metabolic syndrome, sirtuin 1

## Abstract

HIV-infected individuals on chronic use of highly active antiretroviral therapy (HAART) are more likely to develop adipose tissue and metabolic disorders, such as lipodystrophy (LD) and metabolic syndrome (MetS). The development of these phenotypes is known to be multifactorial. Thus, variants in genes implicated in adipogenesis and lipid metabolism may increase susceptibility to LD and MetS. Sirtuin 1 (*SIRT1*) may influence the outcome of these disturbances due to its role in the regulation of transcription factors involved in energy regulation. Therefore, we genotyped four polymorphisms located in *SIRT1* (rs2273773 T>C, rs12413112 G>A, rs7895833 A>G, rs12049646 T>C) in 832 HIV-infected patients receiving HAART by real-time polymerase chain reaction. The prevalence of LD was 55.8% and MetS was 35.3%. Lipoatrophy was the most prevalent subtype in all samples (38.0%) and showed significant difference between white and non-white individuals (*P* = 0.002). None of the genetic variants investigated in *SIRT1* was associated with LD and MetS. White individuals and those in longer time of HAART use were more likely to develop LD. We concluded that these *SIRT1* polymorphisms are not predictive factors to the development of lipodystrophy and metabolic syndrome in HIV-infected individuals from Brazil.

## Introduction

The acquired immunodeficiency syndrome (AIDS), caused by the human immunodeficiency virus (HIV), is globally recognized as an epidemiological phenomenon. Currently, approximately 37 million people are living with HIV (PLHIV) in the world (UNAIDS, 2017). Highly active antiretroviral therapy (HAART) has been shown to improve life quality of HIV-infected individuals, since it completely or almost completely inhibits viral replication, improves the immune system and decreases the incidence of opportunistic infections (Nijhawan, 2012).

It has been shown that HIV-infected individuals in regular use of HAART are at increased risk of developing a variety of metabolic disorders and fat redistribution (Cunha *et al.*, 2015). The latter is known as lipodystrophy syndrome (LD) and can be present as the following subtypes: lipoatrophy (LA), which consists of peripheral subcutaneous fat loss; lipohypertrophy (LH), when there is adipose tissue accumulation, mainly central fat gain. It is also possible, to observe both phenotypes simultaneously, namely mixed lipodystrophy (Guaraldi *et al.*, 2013). LD prevalence is highly variable among studies due to the lack of consensus regarding its definition, diagnostic methods, genetic background, antiretroviral (ARV) drugs scheme, and lifestyle factors (Alves, 2014).

LD can be accompanied by insulin resistance and dyslipidemia. These changes are predictive to the development of metabolic syndrome (MetS), characterized by a set of cardiovascular disease risk factors (CVD), including hypertriglyceridemia, decreased high-density lipoprotein cholesterol levels (HDL-c), and central obesity (Swami, 2016). The prevalence of MetS among HIV-infected patients is also variable among studies (Calza *et al.*, 2017).

The exact mechanism subjacent to LD and MetS development is not elucidated and is believed to be multifactorial (Sacilotto *et al.*, 2017). We have shown evidence that genetic variants in candidate genes involved in adipogenesis and lipid metabolism may be predictors of the occurrence of these metabolic and anthropometric disorders (Gasparotto *et al.*, 2012; Lazzaretti *et al.*, 2013).

The protein silent information regulator 2 (Sir2) regulates aging and lifespan through caloric restriction in *Saccharomyces cerevisiae*, *Caenorhabditis elegans* and *Drosophila melanogaster* (Chen and Guarente, 2007). The enzymatic activity of Sir2 is described as a nicotinamide adenine dinucleotide (NAD+)-dependent deacetylation reaction and relates to the detection of cellular energetic state (nutrients). This protein is highly conserved in several species, from bacteria to mammals (Chen and Guarente, 2007), and presents orthologous genes in higher eukaryotes, in which they are known as sirtuins - *SIRT*s (Kitada *et al.*, 2013).

In mammals, the sirtuin family is comprised by seven proteins (SIRT1-7). Sirtuin 1 (SIRT1), encoded by the *SIRT1* gene (10q21.3), homologous to yeast Sir2, is the best characterized member of class III histone deacetylases (HDACs) (Haigis and Sinclair, 2010). *SIRT1* exerts important biological functions (Lomb, 2010), especially its deacetylase activity in histones favoring heterochromatin formation which, in turn, represses various transcription factors and target proteins involved in lipid and energy regulation, that play roles in adipogenesis and mobilization of fat in white adipose tissue (Clark *et al.*, 2012).

So far, studies have shown a relationship between *SIRT1* and the HIV virus, due to its ability to modulate viral replication: *SIRT1* recycles HIV-Tat protein, which is critical for transcriptional activation of HIV-1 provirus and induces T-cell hyperactivation (Pagans *et al.*, 2005; Kwon *et al.*, 2009; Zhang e Wu, 2009; Pinzone *et al.*, 2013; Shirakawa *et al.*, 2013). However, studies investigating the association of genetic variants on *SIRT1* and metabolic disorders in HIV-infected individuals under HAART have not been found. Therefore, the aim of the present study was to investigate the association between four single nucleotide polymorphisms (SNPs) in *SIRT1* gene (two in the promoter region (rs7895833 A> G and 12049646 T> C), one in intron 4 (rs12413112 G> A) and one in exon 5 (rs2273773 T> C) with LD and MetS phenotypes in PLHIV on regular HAART.

## Material and Methods

### Subjects

This cross-sectional study was conducted with 832 consecutive HIV-infected patients recruited from three government-funded reference centers in the southernmost state of Brazil, Rio Grande do Sul (HIV/AIDS Ambulatory Unit of Hospital de Clínicas from Porto Alegre/RS, HIV Ambulatory Care of Hospital Universitário Dr. Miguel Riet Correa Jr. from Rio Grande/RS, and HIV/AIDS Specialized Assistance Service from Pelotas/RS). Subjects were recruited from March 2006 to November 2008, and from October 2016 to June 2018. All participants were residents of each city or the surrounding region where they were recruited. The enrolled subjects were older than 18 years old, in regular use of HAART for at least 12 months, using two nucleoside reverse transcriptase inhibitors - NRTIs (tenofovir or zidovudine plus lamivudine) and one non-nucleoside reverse transcriptase inhibitor - NNRTI (efavirenz) or protease inhibitor - PI (lopinavir and atazanavir with and without ritonavir), and with viral load below the detection limit of test (50 copies/mL). Pregnant women and patients with neurological illness that impaired the understanding of the study were excluded.

Demographic and lifestyle characteristics were obtained through interviews. Particularities about HIV infection and treatment (time of diagnosis, current ARVs and use of other drugs), as well as other clinical information, were obtained from medical records. Patients were classified phenotypically by the interviewer as white and non-white individuals, as discussed in previous works by our group (Lazzaretti *et al.*, 2013; Castilhos *et al.*, 2015).

### Ethical considerations

This research was approved by the Research Ethics Committees from all centers above mentioned and from the Federal University of Health Sciences of Porto Alegre. All participants signed an informed consent statement before assenting to participate in this study.

### Biochemical and anthropometric parameters

Lipid profile determinations (triglycerides, total cholesterol, and high-density lipoprotein (HDL) cholesterol), fasting serum glucose and viral load were part of patients’ routine care. Physical examination was evaluated through weight (kilograms), height (meters), waist circumference (centimeters) and seven skinfolds by the attending nutritionist or physician. To assess total subcutaneous fat, the sum of skinfolds was grouped into three categories: limb subcutaneous fat (LSF): biceps, triceps and calf folds; central subcutaneous fat (CSF): subscapular, axillary, suprailiac and abdominal folds; and total subcutaneous fat (TSF) was the sum of LSF and CSF (Florindo *et al.*, 2004). Body mass index (BMI) was calculated using the following formula: BMI = weight in kilograms/(height in meters)^2^. LD diagnosis was performed by the attending physician at each center and patients were classified as follows: without LD; lipoatrophy (LA), when fat reduction in the face, arms and/or legs, thin buttocks and venous prominence were observed; lipohypertrophy (LH) was identified by abdominal fat accumulation, gynecomastia in men or increased breasts in women, lipomas through the body or in the dorsocervical region (buffalo hump); a mixed pattern occurred when at least one feature of LA and LH were present together (Alves, Brites e Sprinz, 2014). Metabolic syndrome (MetS) classification was based on International Diabetes Federation (IDF) consensus (IDF, 2006) as waist perimeter ≥ 94 cm in male or ≥ 80 cm in female, plus any two of the following four factors: serum triglycerides ≥ 150 mg/dL or treatment for this lipid abnormality; HDL-cholesterol < 40 mg/dL in males and < 50 mg/dL in females, or drug treatment for reduced HDL-cholesterol; systolic blood pressure ≥ 130 or diastolic blood pressure ≥ 85 mm Hg (or hypertension treatment); fasting plasma glucose ≥ 100mg/dL or previously diagnosed type 2 diabetes (T2D).

### Molecular analysis

Blood samples were collected and sent to the Molecular Biology Laboratory for DNA extraction from peripheral leukocytes using a high salt concentration precipitation standard method. Four polymorphisms in *SIRT1, rs7895833 A>G (promoter region), 12049646 T>C (promoter region), rs12413112 G>A (intron 4), and rs2273773 T>C (exon 5, synonymous, Leu>Leu) were genotyped by real-time polymerase chain reaction (PCR), using primers flanking the target regions coupled to allele discrimination with hydrolysis probes marked with different fluorophores (TaqMan; Applied Biosystems, Foster City, CA). Appropriate negative and positive controls, with different genotypes to each SNP, were used in all the analyses. Although these SIRT1* SNPs have been shown to be in high linkage disequilibrium, the variants analyzed herein were included due to previous reports of associations with BMI in European-derived populations, since no reports of association studies with lipodystrophy in HIV-infected patients were found (Kurylowicz, 2016). Furthermore, little evidence regarding functionality of SNPs in the *SIRT1* gene is available. One study performed by Zarrabeitia *et al.* (2012), suggested that the rs12049646 C-allele had stronger binding ability to nuclear proteins than the T-allele.

### Statistical analysis

A chi-squared test was used to assess the agreement of genotype frequencies with those expected under Hardy-Weinberg equilibrium.

Chi-squared tests were also used to compare categorical variables among ethnicity and lipodystrophy subgroups. When significant differences were found, the partition of chi-squared was performed and adjusted residuals were shown. Anthropometric and demographic variables were compared between ethnicities using *T*-tests for independent samples when normally distributed. To estimate the contribution of each genotypes to LD, LA and LH phenotypes, separately, and MetS, we used Poisson regression with robust variance due to its increased accuracy for analysis of cross-sectional studies with frequent binary outcomes (Barros and Hirakata, 2003). Data analyses were performed with SPSS version 22.0 for Windows software (IBM, Armonk, NY). All tests were two-sided and the differences were considered significant when *P* < 0.05.

## Results

### Characteristics of the participants

Table 1 summarizes the main demographic, clinical and anthropometric characteristics of the 832 HIV-infected subjects enrolled in this research. More than half the sample were males (55.3%). Most of the individuals (59.5%) were phenotypically characterized as white (European ancestry) and 40.5% were non-white individuals (African ancestry); and their mean age was 43.3 ± 10.0 years. The median of HAART duration was 66.0 months. Nearly all patients were on at least three ART drugs, comprising 96.6% using NRTIs, 51.3% on NNRTIs, while 50.8% were receiving PIs. Diabetes mellitus (DM) showed a prevalence of 7.6% of patients and MetS of 35.3%.

**Table 1:** Main demographic, clinical and anthropometric characteristics of 832 HIV-infected individuals on HAART.

Characteristics	HIV-infected patients (n = 832)	White individuals (n = 495)	Non-white individuals (n = 337)	*P* (White vs. Non-white individuals)
Demographic				
Age, years	43.3 ± 10.0	43.8 ± 10.2	42.6 ± 9.72	0.084^a^
Male sex, % (n)	55.3 (460)	57.2 (263)	42.8 (197)	0.148^b^
Clinical				
HAART duration (months), median [IQR]	66.0 [33.0 – 109.0]	69.0 [35.0 – 113.0]	60.0 [30.0 – 107.0]	0.024^c^
PI users, % (n)	50.8 (423)	62.9 (266)	37.1 (157)	0.051^b^
NNRTI users, % (n)	51.3 (427)	56.4 (241)	43.6 (186)	0.076^b^
Diabetes mellitus, % (n)	7.6 (56)^1^	8.0 (37)	7.2 (21)	0.797^b^
Metabolic syndrome, % (n)	35.3 (294)	37.6 (186)	32.0 (108)	0.118^b^
Anthropometric				
BMI (kg/m^2^)	25.2 ± 4.6	25.6 ± 4.9	24.8 ± 4.2	0.025^a^
Lipodystrophy, % (n)	55.8 (461)^2^	63.2 (313)	43.9 (148)	< 0.001^b^
Lipoatrophy subtype, % (n)	38.0 (175)^2^	33.2 (104)	47.9 (71)	0.002^d^
Lipohypertrophy subtype, % (n)	25.6 (118)^2^	28.1 (88)	20.3 (30)	0.072^d^
Mixed subtype, % (n)	36.4 (168)^2^	38.7 (121)	31.8 (47)	0.151^d^

Data are mean ± SD or percentage; ^a^Independent samples *T*-test; ^b^Pearson chi-square with continuity correction; ^c^Mann-Whitney’s U non-parametric test; ^d^*P* from adjusted residuals from chi-square; ^1^Data available for 733 individuals; ^2^Data available for 826 individuals; ^3^Data available for 716 individuals.

The mean BMI in our sample was 25.2 ± 4.6 kg/m^2^ and the prevalence of LD was 55.8%. Among LD patients, the most prevalent subtype was LA (38.0%), followed by mixed pattern (36.4%) and LH (25.6%).

LD prevalence was higher in white (63.2%) than in non-white individuals (43.9%; *P* < 0.001, Table 1). When we compared prevalence of LD subtypes between both ethnic groups, we observed that the mixed subtype was the most prevalent in white individuals (38.7%), while LA was the most prevalent in non-white individuals (47.9%). LH prevalence was apparently higher in white than non-white individuals with no statistical significance (28.1% versus 20.3%, respectively, *P* = 0.072). The LA subtype presented a frequency of 47.9% in non-white individuals and 33.2% in white individuals (*P* = 0.002). The mixed phenotype did not present statistical significance between the two ethnic groups (*P* = 0.151).

### Allele and genotype frequencies

Minor allele frequencies of *SIRT1* polymorphisms analyzed in our study were: rs2273773 (T>C): 0.11; rs7895833 (A>G): 0.25; rs12049646 (T>C): 0.09 and rs12413112 (G>A): 0.11 and are close to those found by 1000 Genomes Project Phase 3 (available at www.ensembl.org) in European and African populations. However, the rs2273773 and rs7895833 genotype frequencies were not distributed according to Hardy-Weinberg equilibrium in our sample. The observed genotype frequencies for rs2273773 were: TT = 636; TC = 140 and CC = 18, while the expected values would be: TT = 629; TC = 155 and CC = 10 (Pearson’s chi-squared test, rs2273773, *P* = 0.0028). The observed frequencies for 7895833 were: AA = 465; AG = 280 and GG = 60, while the expected values would be: AA = 452; AG = 302 and GG = 50 (*P* = 0.0519). No deviation from Hardy-Weinberg equilibrium was found for rs12049646 and rs12413112 variants.

The success rate of the genotyping assay by real-time PCR was 100% to rs12413112, 95.4% to rs7895833; 94.0% to rs2273773 and 92.5% to rs12049646.

Genotype frequencies for all SNPs are shown in Table 2. The SNPs frequencies were compared between white and non-white individuals and did not present statistical difference.

**Table 2:** Genotype frequencies of *SIRT1* rs2273773, rs7895833, rs12049646, rs12413112 genetics variants according to ethnicity in HIV-infected individuals.

*SIRT1* SNPs	Genotype	Frequencies n (%)	Ethnicity		*P* value^1^
			White individuals n (%)	Non-white individuals n (%)	
rs2273773	TT	626 (80.1)	378 (81.6)	248 (77.7)	
	TC	138 (17.6)	75 (16.2)	63 (19.7)	0.405
	CC	18 (2.3)	10 (2.2)	8 (2.6)	
rs7895833	AA	459 (57.8)	272 (57.3)	187 (58.6)	
	AG	277 (34.9)	171 (36.0)	106 (33.2)	0.606
	GG	58 (7.3)	32 (6.7)	26 (8.2)	
rs12049646	TT	645 (83.8)	382 (84.0)	263 (83.5)	
	TC	117 (15.2)	67 (14.7)	50 (15.9)	0.605
	CC	8 (1.0)	6 (1.3)	2 (0.6)	
rs12413112	GG	662 (79.6)	385 (77.8)	277 (82.2)	
	GA	162 (19.5)	103 (20.8)	59 (17.5)	0.121
	AA	8 (1.0)	7 (1.4)	1 (0.3)	

1 Pearson χ^2^ test; number of genotyped individuals: rs2273773 = 782; rs7895833 = 794, rs12049646 = 770; rs12413112 = 832.

### Association analyses between SIRT1 polymorphism and lipodystrophy and metabolic syndrome

The genotype frequencies for the *SIRT1* polymorphisms were compared in individuals without LD and presenting the three LD subtypes separately (Table 3). No significant differences in variants frequencies among these subgroups were observed.

**Table 3:** Comparison of genotype frequencies of rs2273773, rs7895833, rs12049646, rs12413112 genetics variants in *SIRT1* among lipodystrophy subtypes.

*SIRT1* SNPs	Genotype	No LD % (n)	LA % (n)	LH % (n)	MIXED % (n)	*P*
rs2273773	TT	78.1 (268)	80.2 (130)	77.7 (87)	86.2 (137)	0.427^2^
	TC	19.5 (67)	17.9 (29)	18.8 (21)	12.6 (20)	
	CC	2.3 (8)	1.9 (3)	3.6 (4)	1.3 (2)	
rs7895833	AA	55.5 (192)	62.4 (103)	57.0 (65)	58.9 (96)	0.533^1^
	AG	36.1 (125)	30.9 (51)	34.2 (39)	36.8 (60)	
	GG	8.4 (29)	6.7 (11)	8.8 (10)	4.3 (7)	
rs12049646	TT	82.2 (282)	85.7 (138)	81.1 (86)	87.7 (136)	0.570^2^
	TC	16.9 (58)	13.7 (22)	17.9 (19)	11.0 (17)	
	CC	0.9 (3)	0.6 (1)	0.9 (1)	1.3 (2)	
rs12413112	GG	80.8 (295)	80.6 (141)	79.7 (94)	75.6 (127)	0.197^2^
	GA	18.9 (69)	18.9 (33)	19.5 (23)	21.4 (36)	
	AA	0.3 (1)	0.6 (1)	0.8 (1)	3.0 (5)	

1 Pearson *χ* ^2^ test

2 Fisher’s Exact Test; LD, lipodystrophy; LA, lipoatrophy; LH, lipohypertrophy.

Next, we used Poisson regression multivariate analyses to evaluate if the *SIRT1* genetic variants adjusted by biodemographic variables could be predictors to MetS (Table 4) and LD development (Table 5). Regarding the polymorphisms, none was a significant contributor for both phenotypes. The same Poisson regression model was tested on LA and LH separately (Table 6) and we found identical results, with no significant association of SNPs in the *SIRT1* gene and risk of different LD subphenotypes development.

**Table 4:** Poisson regression model and predictive variables for metabolic syndrome (MetS) development in HIV-infected individuals on HAART.

Outcome	Variables	PR	95% CI	*P* value
Metabolic syndrome	Gender (female)	0.95	0.74 – 1.21	0.664
	Age (years)	1.03	1.01 – 1.04	< 0.001
	HAART duration (months)	1.00	1.00 – 1.00	0.976
	Ethnic group (white individuals)	1.09	0.85 – 1.41	0.504
	rs2273773 (C - carriers)	1.01	0.65 – 1.59	0.954
	rs7895833 (G - carriers)	0.98	0.74 – 1.30	0.887
	rs12049646 (C - carriers)	1.11	0.71 – 1.74	0.643
	rs12413112 (A -carriers)	0.85	0.62 – 1.17	0.314
Complete model				*P =* 0.006

PR, prevalence ratio, 95% CI; 95% confidence interval.

**Table 5:** Poisson regression model and predictive variables for lipodystrophy development in HIV-infected individuals on HAART.

Outcome	Variables	PR	95% CI	*P* value
Lipodystrophy	Gender (female)	0.89	0.73 – 1.09	0.259
	Age (years)	1.01	1.00 – 1.02	0.054
	HAART duration (months)	1.00	1.00 - 100	< 0.001
	Ethnic group (white individuals)	1.36	1.10 – 1.68	0.004
	rs2273773 (C - carriers)	0.99	0.69 – 1.42	0.949
	rs7895833 (G - carriers)	0.97	0.77 – 1.22	0.797
	rs12049646 (C - carriers)	0.93	0.64 - 1.34	0.700
	rs12413112 (A -carriers)	0.98	0.77 – 1.24	0.841
Complete model				*P <* 0.001

PR, prevalence ratio, 95% CI; 95% confidence interval.

**Table 6:** Poisson regression model and predictive variables for lipoatrophy and lipohypertrophy development in HIV-infected individuals on HAART.

Outcome	Variables	PR	95% CI	*P* value
Lipoatrophy	Gender (female)	1.00	0.80 – 1.26	0.982
	Age (years)	1.01	1.00 – 1.03	0.023
	HAART duration (months)	1.00	1.00 – 1.01	< 0.001
	Ethnic group (white individuals)	1.35	1.06 – 1.71	0.016
	rs2273773 (C - carriers)	0.94	0.60 – 1.47	0.798
	rs7895833 (G - carriers)	0.97	0.75 – 1.26	0.812
	rs12049646 (C - carriers)	0.91	0.58 – 1.44	0.694
	rs12413112 (A -carriers)	0.97	0.74 – 1.28	0.842
Complete model				*P* < 0.001
Lipohypertrophy	Gender (female)	0.72	0.56 – 0.93	0.010
	Age (years)	1.01	1.00 – 1.02	0.173
	HAART duration (months)	1.00	1.00 – 1.01	< 0.001
	Ethnic group (white individuals)	1.66	1.25 – 2.20	< 0.001
	rs2273773 (C - carriers)	0.91	0.58 – 1.44	0.693
	rs7895833 (G - carriers)	1.04	0.79 – 1.39	0.765
	rs12049646 (C - carriers)	0.96	0.61 – 1.54	0.880
	rs12413112 (A -carriers)	1.01	0.74 – 1.36	0.968
Complete model				*P* < 0.001

PR, prevalence ratio; 95% CI, 95% confidence interval.

Nevertheless, the analyses with biodemographic variables showed that age (years), HAART duration (months) and white individuals are predictors to a higher risk to LD development. The same predictive variables were significant predictors of LA, particularly. For LH, in addition to HAART duration and ethnicity (white individuals), the female gender also contributed to the occurrence of this phenotype. Regarding MetS, only higher age contributed significantly to this metabolic disorder.

## Discussion

In this study, we analyzed the association between variants in *SIRT1* gene with lipodystrophy and metabolic syndrome occurrence in HIV-infected patients on HAART. To our knowledge, this is the first study to investigate this relationship and our results suggest that genotypes of rs12413112, rs7895833, rs2273773 and rs12049646 polymorphisms in *SIRT1* are not predictor factors of metabolic disorders in Brazilian patients infected by HIV using regular antiretroviral regimen.

The Brazilian population presents a unique setting to study HIV-infected patients due to a highly successful program of antiretroviral therapy distribution to all infected individuals, free of charge and according to the same guidelines all over the country. These patients are mostly treated in reference centers maintained by the Brazilian Ministry of Health, which favors the recruitment of a higher number of participants as presented herein. Furthermore, this work includes a high number of women and individuals of African ancestry, mainly neglected in other studies.

Metabolic balance results from a complex interaction between calorie intake, expenditure and energy storage, intimately orchestrated by many signaling pathways and genetic expression. The physiological and molecular mechanisms underlying this regulation are variable among individuals. It is widely accepted that genetic background, gender, behavior factors and environmental influences act in the disruption of energy homeostasis and adipose tissue distribution (van der Klaauw and Farooqi, 2015). In this context, mammalian sirtuin 1 protein (encoded by *SIRT1* gene) has attracted attention into metabolic disorders studies due to its contribution in several physiological processes, including glucose metabolism and adipogenesis. Its yeast orthologue, silent information regulator 2 (encoded by the *SIR2* gene), was able to extend lifespan in lower organisms through prolonged calorie restriction (CR). The positive effects of prolonged CR in health are due its ability to delay the onset of age-related diseases (Dali-Youcef *et al.*, 2007).

Some studies have shown that single nucleotide polymorphisms (SNPs) in *SIRT1* can change its expression and activity, leading to individual susceptibility to obesity and related metabolic disturbances in non-HIV infected subjects (Kurylowicz, 2016). In a large Dutch cohort (n = 3575), subjects bearing the TC genotype of rs2273773 T>C presented a BMI 0.5 kg/m^2^ higher than TT homozygotes. In another study with Japanese healthy subjects, the rs7895833 G>A and rs2273773 T>C SNPs were associated with laboratory (biochemical, metabolic and lipid) and anthropometric parameters. Men carriers of the A allele of rs7895833, compared to GG homozygotes, had a higher body fat ratio and BMI. Body fat ratio was also higher in men TT homozygotes for rs2273773, as well as hyperglycemia (Shimoyama *et al.*, 2011). Our results do not support these findings in HIV-infected individuals and suggest that the *SIRT1* gene may not be involved in the etiology of lipodystrophy in these patients. Meneguette *et al.* (2016) did not find differences in genotype and allele frequency of rs7895833 A>G SNP *SIRT1* between Brazilian adult individuals with metabolic syndrome (MetS) and those not presenting it (*P* = 0.24). The minor allele-G frequency was 0.280 in subjects with MetS and 0.265 in non-MetS (Meneguette *et al.*, 2016).

In the present study, the genotype frequencies of rs12413112, rs7895833, rs2273773 and rs12049646 polymorphisms in *SIRT1* gene were compared between LD subtypes and no-LD patients, but no significant differences were detected. Next, we used Poisson regression multivariate analyses to evaluate the contribution of demographic and clinical variables and common allele SNPs to MetS and LD development. Again, none of the genetic polymorphisms were predictive to the occurrence of this phenotypes. We observed that higher age (years) was a risk factor to MetS. Regarding LD and LA subtypes development, age (years), HAART duration (months), and ethnic group (white individuals) were predictive to lipodystrophy development. However, for LH subtype we found that HAART duration (months), ethnic group (white individuals), and female gender were significantly associated with this outcome.

It has already been established that sex hormones influence fat deposition pattern, as well as adipose tissue function (Palmer and Clegg, 2015) and endocrine diseases (Lauretta *et al.*, 2018). Galli *et al.* (2003) reported that in HIV-infected patients abdominal and breast fat accumulation, rather than subcutaneous fat wasting, has been shown to be the dominant manifestation in women on HAART in addition to hypertriglyceridemia. Regarding ethnicity, white subjects were more likely to develop LD than non-whites. However, while in white individuals the lipohypertrophy and mixed subtypes were more prevalent than in non-whites, the predominant pattern in non-whites was lipoatrophy (Galli *et al.*, 2003). Andany *et al.* (2011), studying HIV-infected subjects from Canada, verified that white individuals had a greater chance of presenting body fat changes, especially central lipohypertrophy and peripheral lipoatrophy in males, but not in females, when compared to their non-white counterparts (Andany *et al.*, 2011). Although their population is hardly comparable to that studied herein regarding ethnicity, theirs and our results reinforce the idea that lipoatrophy and lipohypertrophy phenotypes are singular entities, with distinct multifactorial underlying etiology.

The heterogeneity within and among different regions of Brazil is due to three ancestral roots: Amerindians, Europeans and sub-Saharan Africans (Suarez-Kurtz, 2010). Individuals from the Rio Grande do Sul state phenotypically classified as white presented an estimate of 85% of European ancestry according to in/del genetic markers, while those classified as non-white (black and brown) had only 45% of African ancestry. Despite this admixture of our population, the genomic ancestry of Brazilians is more uniform than thought (Pena *et al.*, 2011). This needs to be considered in interpretations and extrapolations of our data to other regions of the world.

The prevalence of LD and MetS in PLHIV is widely variable due to study design, sample heterogeneity and criteria used to diagnosis (Loonam and Mullen, 2012; Alves, 2014). LD is a complex syndrome, which requires an accurate clinical view, patient report and anthropometric evaluation, classifying it as a unique entity (Koethe, 2017). Similarly, the pathophysiology of MetS in HIV-infected population may include HIV itself, ARVs drugs chronic use, and persistent inflammation (Srinivasa and Grinspoon, 2014). In our study, we found a prevalence of 55.8% of LD and of 35.5% of MetS. Although more than half of our sample had lipodystrophy, glycemic, and lipid disturbances, they did not always follow this phenotype. These findings are corroborated by Sacilotto *et al.* (2017) who did not find statistical significant differences in metabolic profile alterations among lipodystrophy subtypes (Sacilotto *et al.*, 2017).

In the present study, the genotypic frequencies of the SNPs rs2273773 T>C and rs7895833 A>G in *SIRT1* gene were not in accordance to those expected under Hardy-Weinberg equilibrium. We considered some possibilities: first, recruitment bias. However, the other two polymorphisms (rs12049646 T>C and rs12413112 G>A), and several other SNPs previously investigated by our group in this same sample, were following Hardy-Weinberg equilibrium. Second, errors in the genotyping assay by real-time PCR. Nonetheless, appropriate controls were used in each batch, and the used probes were clearly able to discriminate genotypes. Third, the Hardy-Weinberg deviation occurred by chance, because the differences observed between expected and observed genotype frequencies were small. Estimates of European contribution for the Rio Grande do Sul population vary from 81% (Callegari-Jacques *et al.*, 2003) to 94% (Santos *et al.*, 2009). Our sample was composed by 41% of individuals classified as brown or black, which is a much higher proportion than the one found in the general population of the state. We believe this may be due to the fact that users of the public health system in Brazil come from the extracts with lower income, where the African derived individuals are concentrated. Although, this does not seem to be an issue for the results found, it could explain the deviation from Hardy-Weinberg equilibrium observed in SNPs in this work.

It is known that HIV infection causes immune activation and persistent inflammation condition via cytokine production of latently infected CD4+ T cells in adipose tissue. The exacerbated local cytokines consequently these resting cells and promote viral shedding, consequently, hence, increases proinflammatory mediators from adipocytes, thus releasing the viral protein R (Vpr) and Trans-activator of transcription (Tat) HIV-proteins (Koethe, 2017). Tat is a crucial regulatory protein with a key role on viral transcription and HIV pathogenesis (de Goede *et al.*, 2015). Experimental studies showed that preadipocytes exposed to Tat protein do not achieve maturation due to decreased mRNA of adiponectin, peroxisome proliferator-activated receptor gamma (*PPARG*), and glucose transporter type 4 (*GLUT4*), while they increase proinflammatory cytokine and reduce glucose uptake (Koethe, 2017). The relationship of *SIRT1* and HIV-infection has already been described. The *SIRT1* is able to promote deacetylation and acetylation cycles that regulated HIV transcription, acting as transcriptional coactivator and inducing T-cells hyperactivation (Pagans *et al.*, 2005; Kwon *et al.*, 2009). Also, Tat is a substrate to SIRT1 enzymatic activity protein, through binding in the active center of deacetylase domain, leading to inhibition of SIRT1 function (Zhang and Wu, 2009).

Once suppressed, *SIRT1* induces the acetylation of p53 that can arrest cell cycle in HIV infected cells and high expression of p21 and BAX (Thakur *et al.*, 2012). This information could explain why we did not find any association between variants in *SIRT1* with metabolic imbalance and anthropometric disturbances in our sample. Thus, we suggest that HIV virus, and infection itself, should affect in other ways the *SIRT1* activity, besides its suppression by Tat-HIV protein. Also, the ARV drugs may impair *SIRT1* expression, and their target genes, in a manner that has not been identified yet.

The present study has some limitations. First, this is a cross-sectional study. So, it is only able to find associations and not causality. Second, treatment heterogeneity in our sample. Each antiretroviral drug may act on a different molecular level in *SIRT1* expression. We did not investigate this possibility. As far as we know, no study has analyzed this influence at the cellular level yet. Thirdly, the lack of association among the SNPs investigated in this study and LD and MetS occurrence may be due to non-genotyping of causative variant within *SIRT1* gene. Fourthly, the lipodystrophy syndrome has heterogeneous character, in addition to absence of a methodological guideline for diagnosis; thus, there is some subjectivity in classification.

In conclusion, our results show that the genetic variants rs12413112, rs7895833, rs2273773 and rs12049646 in *SIRT1* gene are not predictive factors of higher risk for LD and MetS among HIV-infected population on regular HAART from Brazil. To our knowledge, the present study is a pioneer investigation of this relationship in the population infected by HIV. Further experimental research is required to elucidate the gaps concerning the mechanism of action of HIV virus and ARV drugs on *SIRT1* activity as deacetylase of genes involved in lipid metabolism and adipogenesis in PLHIV.
